# Feasibility of Escape Room Simulation in Community Health Nursing Education: A Quasi-Experimental Study of Student Perceptions of Competence and Confidence

**DOI:** 10.3390/nursrep16060189

**Published:** 2026-05-29

**Authors:** Lufei Young, Xi Ning, Yinghao Pan, Tiffany Jackson, Meredith Troutman-Jordan

**Affiliations:** 1 School of Nursing, University of North Carolina at Charlotte, Charlotte, NC 28223, USA; tjack109@charlotte.edu (T.J.); mflood@charlotte.edu (M.T.-J.); 2 Department of Statistics, Colby College, Waterville, ME 04901, USA; xning@colby.edu; 3 Department of Mathematics and Statistics, University of North Carolina at Charlotte, Charlotte, NC 28223, USA; ypan8@charlotte.edu

**Keywords:** escape room simulation, competence, confidence, community health nursing, individual variability modeling

## Abstract

**Background/Objectives:** Community health nursing (CHN) clinical experiences are essential for preparing undergraduate nursing students to deliver safe and effective home-based care; however, access to placements is often limited. Escape room simulation (ERS) has been used in nursing education, yet its feasibility in CHN remains underexplored. This exploratory study aimed to examine the feasibility of using ERS in undergraduate CHN education by evaluating students’ perceived competence, confidence, and learning experiences following participation in the simulation. **Methods:** A quasi-experimental, single-group pre–post design was conducted with 56 undergraduate nursing students. Data were collected at three time points: prior to the simulation, immediately after, and ten weeks post-intervention. Measures included standardized assessments of CHN competence and confidence, as well as student perceptions of the simulation experience. Linear mixed-effects modeling was used to examine changes over time. **Results:** Significant improvements were observed in both competence (t = 6.413, *p* < 0.001) and confidence (t = 8.142, *p* < 0.001) following the simulation, with gains maintained at ten weeks. Variability in competence decreased across participants over time, while confidence gains varied individually, with larger improvements among participants with lower baseline scores. Participants reported high levels of satisfaction with the simulation despite limited prior exposure. **Conclusions:** ERS was associated with improvements in undergraduate nursing students’ perceived competence and confidence, suggesting that it may represent a feasible and acceptable supplemental educational strategy for undergraduate CHN education. Additional research is needed to better evaluate the educational impact and underlying mechanisms of ERS in CHN education, and to support the development of evidence-based ERS approaches that enhance student preparedness for community-based nursing practice in settings where clinical placements are limited.

## 1. Introduction

Clinical practice is a cornerstone of nursing education, providing students with essential opportunities to develop the skills, confidence, and competence required for safe and effective patient care. However, access to high-quality clinical placements has become increasingly limited due to multiple systemic challenges, including shortages and high turnover of clinical preceptors, overstretched healthcare systems, competition among academic programs, and geographic constraints. These limitations are particularly pronounced in community health settings, where students often have reduced exposure to underserved and marginalized populations. As a result, nursing graduates may be insufficiently prepared to deliver culturally responsive care, address health disparities, identify social determinants of health (SDOH), and function effectively in complex, real-world community environments [1]. These challenges also place strain on nursing programs, affecting enrollment capacity, curriculum delivery, and faculty workload, ultimately impacting the quality of nursing education and workforce readiness.

Community health nursing (CHN) education requires students to develop competencies beyond traditional acute care clinical skills, including home and environmental assessment, therapeutic communication, cultural responsiveness, interprofessional collaboration, clinical reasoning, and assessment of psychosocial and socioeconomic factors that influence health outcomes [1]. Unlike hospital-based settings, community health nurses frequently practice independently in patients’ homes and communities where environmental hazards, caregiver burden, food insecurity, transportation barriers, financial strain, social isolation, and limited access to healthcare resources may directly affect patient safety and well-being [2]. However, opportunities for students to encounter these complex situations during traditional clinical placements may be inconsistent or limited, particularly in resource-constrained community settings.

To address these constraints, nursing education has increasingly explored alternative and supplemental strategies to enhance experiential learning. One emerging approach is the use of game-based educational interventions [3,4,5]. Escape room simulations (ERSs) are team-based, game-oriented activities that require students to solve problems collaboratively under time constraints, promoting critical thinking, communication, and decision-making skills [6,7,8]. Evidence across health professions education suggests that ERS can enhance student engagement, motivation, and satisfaction, and may support the development of clinical reasoning and teamwork skills [9,10,11,12,13,14,15,16]. In nursing education, ERS has been associated with improvements in clinical competencies and collaborative problem-solving in a safe, interactive learning environment [17].

ERS may be particularly well suited for CHN education because it allows students to actively engage in realistic community-based scenarios involving home safety assessment, patient interviewing, SDOH screening, medication management, risk identification, prioritization, interdisciplinary problem-solving, and culturally responsive communication within immersive and psychologically safe learning environments. The interactive nature of ERS may also encourage teamwork, self-reflection, and application of clinical judgment while exposing students to socially and culturally complex patient situations that may not consistently occur during routine clinical experiences. Unlike traditional classroom instruction, ERS may provide opportunities for students to simulate independent decision-making and environmental assessment processes commonly required in community-based nursing practice.

Despite these promising findings, the current evidence base remains limited and heterogeneous. Studies vary widely in design, implementation, and outcome measures, making it difficult to draw consistent conclusions about effectiveness [7,18]. While some research supports ERS as effective adjuncts to traditional teaching, others highlight the lack of standardized evaluation and the need for more rigorous study designs. Furthermore, the application of ERS in community health nursing (CHN) education remains underexplored, particularly in relation to long-term outcomes such as sustained competence and confidence.

Given the growing need to prepare nursing students for community-based practice in increasingly complex and resource-constrained environments, as well as the limited evidence regarding the educational impact of ERS in CHN education, we conducted an exploratory study examining the feasibility of using ERS in undergraduate CHN education by evaluating students’ perceived competence, confidence, and learning experiences over time. Rather than determining definitive intervention effects, this study aimed to provide preliminary evidence regarding the feasibility, acceptability, and potential educational application of ERS within CHN education. The findings may help guide future research using more rigorous controlled designs and objective performance-based outcome measures to better evaluate the educational impact and underlying mechanisms of ERS, as well as support the development of evidence-based ERS approaches to enhance student preparedness for community-based nursing practice in settings where clinical placements are limited.

## 2. Materials and Methods

### 2.1. Study Design

A quantitative quasi-experimental, single-group pre–post design was used to evaluate the impact of an escape room simulation (ERS) on undergraduate nursing students’ competence and confidence in community health nursing (CHN) practice (Supplementary Files SI and SII). This design allowed project implementation and data collection without interrupting the existing nursing curriculum [19]. Because the ERS activity was a required graded learning experience integrated within the CHN curriculum, withholding the educational activity from a subgroup of students was not considered educationally appropriate or ethically feasible. Therefore, findings should be interpreted cautiously as exploratory associations rather than definitive intervention effects.

### 2.2. Study Participants and Setting

Participants were a convenience sample of undergraduate nursing participants. The inclusion criteria consisted of undergraduate nursing participants enrolled in the required community health nursing (CHN) course who participated in the escape room simulation (ERS) activity as part of a graded clinical learning experience and agreed to participate in the research data collection component. Participants who declined research participation, withdrew from the course, or did not complete the required study questionnaires were excluded from data analysis. Participants were informed that participation in the research data collection component was voluntary and that declining participation or withdrawing from the study would not affect their course grade, clinical evaluation, or relationship with faculty. Because the ERS activity was integrated into the required CHN curriculum, all enrolled participants participated in the educational activity regardless of research participation status. Written informed consent was obtained before data collection, and the study was approved by the university Institutional Review Board (IRB). A total of fifty-six participants participated in the study. To maintain anonymity and confidentiality, survey responses were collected using de-identified participant codes rather than student names, and all data were stored on password-protected university-approved secure systems accessible only to the research team. Faculty involved in course grading were blinded to study participation and survey responses, and results were reported only in aggregate form to prevent identification of individual participants.

The simulation was conducted in the School of Nursing Simulation Center using apartment-style simulation rooms designed to resemble realistic community home environments. The ERS was used as a formative learning experience rather than a high-stakes summative evaluation. To reduce potential bias and maintain consistency across groups, faculty facilitators followed standardized simulation guides, scoring checklists, pre-briefing instructions, and structured debriefing procedures (Supplementary File SI–SIII). Faculty observers primarily served in non-interventional roles and only intervened if major safety concerns, professionalism concerns, or substantial disruption of the activity occurred (Supplementary File SIII).

### 2.3. Description of the Escape Room Simulation

The escape room simulation (ERS) was originally developed by faculty and researchers with expertise in community health nursing education, simulation, and research, with support from National League for Nursing funding [20,21]. The original simulation was adapted for this study to specifically address core CHN competencies guided by the Quad Council Coalition (QCC) Public Health Nursing Competency Framework [22].

The revised ERS emphasized comprehensive home assessment, therapeutic communication, social determinants of health (SDOH), cultural responsiveness, environmental safety, medication management, caregiver burden, chronic disease management, clinical reasoning, and interdisciplinary problem-solving. The simulation was intentionally designed to reflect the complexity and unpredictability of community-based nursing practice, where nurses frequently encounter overlapping medical, psychosocial, environmental, and socioeconomic concerns while working independently in patients’ homes. Detailed ERS design materials, including the simulation protocol, standardized patient scripts, clue structure, faculty guides, student instructions, and debriefing guides, were provided in the Supplementary Materials (Supplementary File SI–SX).

The International Nursing Association for Clinical Simulation and Learning (INACSL) Healthcare Simulation Standards of Best Practice™ were used to guide the development and implementation of the ERS activities [23]. The simulation incorporated multiple components aligned with best practices in simulation-based education, including structured pre-briefing, psychologically safe orientation, immersive scenario-based learning, standardized patient interaction, environmental assessment, collaborative team activities, faculty observation, and structured reflective debriefing. The ERS was implemented in apartment-style simulation rooms staged with realistic household furnishings, medication bottles, food items, mobility equipment, environmental hazards, written clues, and patient care cues designed to simulate complex community-based nursing situations commonly encountered during home visits (Supplementary File SIII).

Participants were divided into 14 groups of four participants each, and each group participated in approximately 45-min ERS activity. Prior to the ERS activity, participants participated in a structured pre-briefing session that included orientation to the apartment-style simulation environment, learning objectives, workflow expectations, escape room procedures, psychological safety expectations, and team-based communication strategies. Participants were informed that the simulation was designed as a supportive learning experience rather than punitive testing. Faculty emphasized respectful communication, collaborative learning, learner autonomy, and participants’ freedom to ask questions or exit the activity if needed (Supplementary File SIII).

The ERS activity consisted of two integrated stations: (1) standardized patient (SP) interaction (Supplementary File SIV) and (2) comprehensive home assessment. During the SP interaction station, participants interviewed a standardized patient portraying an older adult caregiver from a minority ethnic group who was managing multiple chronic health conditions and socioeconomic stressors. Participants practiced therapeutic communication and conducted a structured assessment using a SDOH structured screening tool designed to identify health and social risks, including chronic disease management challenges, home safety concerns, caregiver burden, financial strain, social support needs, and barriers to healthcare access (Supplementary File SV). During the home assessment station, participants systematically evaluated the simulated apartment, including the living room, kitchen, bedroom, and bathroom, for environmental safety concerns and patient care risks (Table 1). Embedded clues and lockbox challenges required participants to identify trip hazards, fall risks, food insecurity indicators, medication safety problems, accessibility barriers, sanitation concerns, and chronic disease management issues (Supplementary File SVI). To successfully “escape”, teams were required to complete the SP interview, conduct all environmental assessments, correctly identify required clues, demonstrate safe and therapeutic nursing behaviors, and correctly enter the final lockbox code sequence (Supplementary File SVII).

Faculty facilitators used standardized moderator guides to maintain consistency across student groups. Faculty primarily observed student performance and documented communication, assessment, teamwork, and clinical reasoning behaviors for later discussion during debriefing. Faculty did not actively intervene during the simulation unless a major safety concern or breakdown in the activity occurred (Supplementary File SIII). Following completion of the ERS, a 45-min debrief session was moderated by trained faculty using the Debriefing Assessment for Simulation in Healthcare (DASH) guideline to support reflective learning [24]. The debriefing session focused on reflection, teamwork, communication, clinical judgment, cultural responsiveness, SDOH considerations, and application of CHN concepts. Debriefing discussions encouraged participants to reflect on cultural humility, implicit bias awareness, interdisciplinary collaboration, patient-centered communication, and lessons learned from the simulation experience (Supplementary File SVIII).

### 2.4. Measures and Data Collection

Data collections were conducted at three time points to determine the impact of ERS: pre-ERS (T_1_), post-ERS (T_2_), 10 weeks after ERS session (T_3_). The instruments we used for data collection include (1) demographic form; (2) CHN competency checklist; (3) CHN confidence scale; and (4) Escape room experience perception scale. Prior to the ERS activity, the participants completed the demographic form, CHN competency checklist and confidence scale. At the end of the session, participants were asked to complete the CHN competency checklist, confidence scale, and escape room experience perception scale. Finally, 10 weeks after the ERS session, participants completed the CHN competency checklist and confidence scale.

Community Health Nursing (CHN) Competency Checklist is an 18-item checklist that includes the clinic competency skills for community health nursing practice required by the Quad Council Coalition (QCC) of Public Health Nursing Organizations [22]. Participants reported their competency for each skill by responding “Yes” or “No”. Each student’s competency was operationalized as the sum score the number of “Yes”. Because the checklist used dichotomous response options, internal consistency reliability was evaluated using the Kuder–Richardson Formula 20 (KR-20), which is equivalent to Cronbach’s alpha for binary items. The checklist demonstrated good internal consistency at baseline (KR-20 = 0.89).

Community Health Nursing (CHN) Confidence Scale includes 18 items that measure participants’ confidence in performing CHN skills using skills for community health nursing practice. Participants were asked to rate their confidence to perform the listed clinical skills on a 100-point scale, ranging in 10-unit intervals from 0 (“Cannot do”); through intermediate degrees of confidence, 50 (“Moderately certain can do”); to complete confidence, 100 (“Highly certain can do”). The higher sum score indicates greater confidence in practicing community health nursing care. Internal consistency was excellent (Cronbach’s α = 0.95).

Escape Room Experience Perception Scale is a 12-item scale used to assess student perceptions across four domains: enjoyment, feasibility/usefulness, relevance, and effectiveness. Each item was measured on 5-point Likert-type scale ranging from 1 (strongly disagree) to 5 (Strongly agree). The higher sum score indicates the more positive learning experience during escape room simulation. This scale was administered following the conclusion of the escape room simulation session. Internal consistency was excellent (Cronbach’s α = 0.97).

### 2.5. Data Analysis

All analyses were conducted using R studio (version 2023.06.1+524) with a significance level of 0.05. Descriptive analyses were performed to describe the study participants, student competencies, confidence and ERS experiences and perceptions. Frequencies and percentages were used for categorical variables, while means and standard deviations (SD) were used for continuous variables.

To evaluate changes in competency and confidence across the three measurement time points (pre-ERS [T_1_], post-ERS [T_2_], and 10 weeks after the ERS session [T_3_]), linear mixed-effects models were employed to account for the repeated-measures structure of the data. Specifically, a random-intercept model was used, with time included as a fixed effect and participant included as a random intercept to account for within-subject correlations across repeated observations. This modeling approach was selected because repeated observations from the same participant are not statistically independent, and mixed-effects models allow for individual variability in baseline scores while estimating overall time-related changes. The model structure was specified as:

$$Y_{ij}=\mu_{j}+\alpha_{i}+\epsilon_{ij}$$

where $Y_{ij}$ represents competence or confidence for participant i at time j, $\mu_{j}$ represents the fixed effect of time, $\alpha_{i}$ is the random participant effect, and $\epsilon_{ij}$ is the residual error term. Model assumptions were evaluated using residual diagnostics, including residual plots, Q–Q plots, and normality testing of model residuals. Effect sizes were calculated using paired-sample Cohen’s dz to evaluate the practical magnitude of change across time points.

Data visualization, including longitudinal (spaghetti) plots of competence and confidence, was performed using the ggplot2 package in R.

### 2.6. GenAI Use Disclosure

No generative artificial intelligence (GenAI) tools were used in study design, data collection, analysis, or interpretation. GenAI was used only for minor language editing (grammar and clarity), which does not require formal disclosure per journal guidelines.

## 3. Results

### 3.1. Participants Characteristics

A total of 56 full-time participants participated in the ERS activity, and all participants completed data collection at all three time points. Participant characteristics are presented in Table 2. The mean age of the sample was 22 years (SD = 2.795). Participants included 53 females (94.6%) and 3 males (5.4%). Racial demographics included 29 White participants (51.8%), 11 Black participants (19.6%), 7 Hispanic participants (12.5%), 7 Asian participants (12.5%), 1 Middle Eastern participant (1.8%), and 1 multiracial participant (1.8%). Most participants reported English as their primary language (*n* = 41, 73.2%), and 12 participants (21.4%) were multilingual. The average years of education was 16 years (SD = 2.084). All participants were full-time participants. Participants reported working an average of 10.88 h per week (SD = 11.188). Most participants had previous unlicensed nursing work experience (*n* = 46, 82.1%) in various healthcare settings, including acute care (*n* = 19, 33.9%), ambulatory clinics (*n* = 4, 7.1%), nursing homes (*n* = 4, 7.1%), assisted living facilities (*n* = 4, 7.1%), home health (*n* = 4, 7.1%), skilled nursing facilities (*n* = 1, 1.8%), rehabilitation centers (*n* = 1, 1.8%), and multiple healthcare sites (*n* = 8, 14.3%). The average length of employment was 41.22 months (SD = 24.235). Thirteen participants (23.2%) reported previous escape room experience, with an average of 2.77 h of prior experience (SD = 2.713).

### 3.2. Impact of ERS on CHN Competency, Confidence, and ERS Experience

The scores on the Community Health Nursing (CHN) Competency Checklist and Confidence Scale were analyzed using data collected at pre-ERS (T_1_), post-ERS (T_2_), and 10 weeks after the ERS activity (T_3_). Scores on the Escape Room Experience Perception Scale were collected immediately after the ERS session at T_2_. Table 3 presents the minimum, median, mean, maximum, standard deviation, and effect sizes (Cohen’s d) for CHN competence, confidence, and perceived ERS experience across the measurement time points.

The mean competence score increased from 14.98 (SD = 3.88) at T_1_ to 17.39 (SD = 1.27) at T_2_ and 17.85 (SD = 0.62) at T_3_. Cohen’s d effect sizes for competence were 0.810 at T2 and 0.799 at T_3_ relative to baseline.

The mean confidence score increased from 1295.56 (SD = 276.08) at T_1_ to 1535.98 (SD = 204.09) at T_2_ and 1689.45 (SD = 111.26) at T_3_. Cohen’s d effect sizes for confidence were 1.241 at T_2_ and 1.494 at T_3_ relative to baseline.

Standard deviations for both competence and confidence scores decreased across time points. For competence, standard deviations decreased from 3.88 at T_1_ to 1.27 at T_2_ and 0.62 at T_3_. For confidence, standard deviations decreased from 276.08 at T_1_ to 204.09 at T_2_ and 111.26 at T_3_.

The Escape Room Experience Perception Scale was administered immediately after the ERS session at T_2_. Participants reported a mean score of approximately 60 (SD = 8.19).

### 3.3. Individual Growth Plots in CHN Competence and Confidence

**Figure 1:**
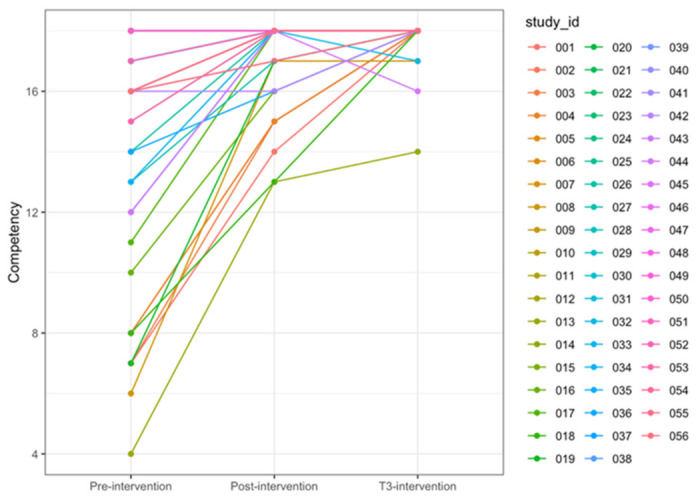
Individual growth plot in competence.

**Figure 2:**
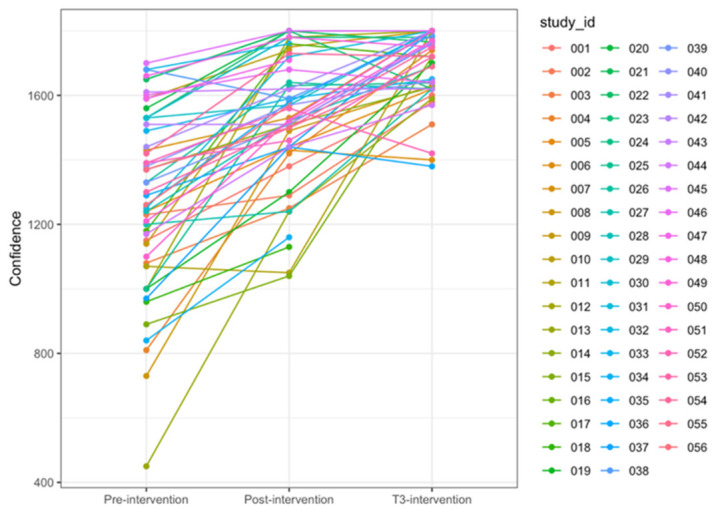
Individual growth plot in confidence.

Figure 1 and Figure 2 present spaghetti plots illustrating individual longitudinal trajectories in CHN competence (Figure 1) and confidence (Figure 2) across the three measurement points: pre-ERS (T1), post-ERS (T2), and 10 weeks following the ERS activity (T3). Each line represents one participant’s scores over time. The x-axis represents the measurement points, and the y-axis represents CHN competence or confidence scores. The plots were generated using the ggplot2 package in R to visualize individual trajectories and variability across participants. In both figures, most participant trajectories demonstrated upward trends from T1 to T2 and T3. Competence trajectories appeared relatively consistent across participants, whereas confidence trajectories demonstrated greater variability across time points. The color variation in the spaghetti plots was used to visually illustrate diversity in longitudinal response patterns and did not represent predefined statistical thresholds or effect sizes.

### 3.4. Inferential Analysis

The final model analyses showed that the participants’ CHN competence scores significantly improved when comparing pre- and post-intervention scores (*t*-statistic = 6.413, *p*-value < 0.001) (Table 4). The estimated average competence scores for pre-ERS (T1), post-ERS (T2), and T3 were 14.982, 17.392, and 17.870, respectively. The 95% confidence intervals for the differences in means (Post − Pre and T3 − Pre) both excluded 0, indicating statistically significant differences. An approximate 95% confidence interval for the standard deviation σ_α ranged from 0.862 to 1.795.

For competence, residual diagnostics indicated some deviation from normality, likely related to the dichotomous scoring structure and ceiling effects at later time points (Shapiro-Wilk W = 0.813, *p* < 0.001). The model explained a moderate proportion of variance (marginal R^2^ = 0.220; conditional R^2^ = 0.460; ICC = 0.308). Effect sizes showed moderate-to-large improvement from T1 to T2 (Cohen’s dz = 0.810) and from T1 to T3 (Cohen’s dz = 0.799).

A statistically significant increase was also noted in participants’ confidence in community health nursing practice after the completion of ERS activity (*t*-statistic = 8.142, *p*-value < 0.001) (Table 5). The estimated average confidence scores at the pre-ERS, post-ERS, and T3 time points are 1290.687, 1535.982, and 1694.177, respectively. A 95% confidence interval for the standard deviation σ_α ranges between 105.966 and 189.578.

For confidence, residual diagnostics were more acceptable, although the Shapiro–Wilk test remained statistically significant (W = 0.972, *p* = 0.002), suggesting minor deviation from normality. The model explained a larger proportion of variance (marginal R^2^ = 0.364; conditional R^2^ = 0.661; ICC = 0.467). Effect sizes showed large practical improvements from T1 to T2 (Cohen’s dz = 1.241) and from T1 to T3 (Cohen’s dz = 1.494).

Sensitivity analyses were subsequently conducted to examine whether demographic and experiential variables influenced the longitudinal outcome trajectories. Variables including previous ERS experience, prior healthcare work experience, age, gender, language status, and work hours were entered as covariates in the mixed linear models. Most demographic variables, including previous ERS experience, age, gender, language status, and work hours, did not demonstrate statistically significant effects on longitudinal changes in competence or confidence (all *p* > 0.05). However, prior healthcare work experience demonstrated a significant association, suggesting that previous clinical exposure may partially contribute to variability in student outcomes. Overall, the primary findings regarding improvements in competence and confidence remained stable after adjustment for these covariates.

## 4. Discussion

This study primarily focused on examining the feasibility of using an escape room simulation (ERS) within undergraduate community health nursing education by evaluating participants’ perceived competence, confidence, and learning experiences over time. Overall, participation in the ERS was associated with improvements in participants’ perceived competence and confidence immediately following the activity, with improvements sustained 10 weeks later. Participants reported high satisfaction with the ERS activities and perceived the experience as helpful and effective in supporting their clinical learning and practice in community health nursing. Participants also reported positive learning experiences related to engagement, teamwork, communication, and active participation. Collectively, these findings suggest that ERS may represent a feasible and acceptable supplemental educational strategy within undergraduate CHN education, particularly in settings where access to traditional community-based clinical placements is limited.

The findings are generally consistent with prior nursing and health professions education literature suggesting that ERS activities may enhance learner engagement, teamwork, motivation, communication, and experiential learning [5,7,18,25]. However, much of the existing literature has focused primarily on learner satisfaction or short-term educational experiences. In contrast, the present study contributes preliminary longitudinal data examining changes in participants’ perceived competence and confidence over time following participation in a CHN-focused ERS activity. Although the current design does not permit causal inferences regarding intervention effectiveness, the sustained improvements observed across measurement points suggest that ERS may have potential educational value warranting further investigation using more rigorous methodologies.

One finding worth noting is that the standard deviations decreased across time points, which may suggest reduced variability in participants’ perceived competence and confidence following the ERS activity. One possible explanation is that students became more aligned in their learning outcomes and self-perceptions over time. However, these findings should be interpreted cautiously, as the reduced variability may also reflect ceiling effects.

Another important observation in this study was the spaghetti plots demonstrated different variability patterns between competence and confidence trajectories over time. Although group-level analyses demonstrated overall increases in both domains, individual confidence trajectories appeared more heterogeneous than competence trajectories. Some participants demonstrated plateaued or downward confidence trajectories despite increases in competence scores, whereas others reported increased confidence with minimal changes in competence. These findings suggest that competence and confidence may represent related but distinct educational constructs that do not necessarily develop uniformly during experiential learning activities.

The differing trajectories observed between competence and confidence may be partially explained by Bandura’s Self-Efficacy Theory, which proposes that perceived confidence in one’s abilities is influenced not only by skill acquisition, but also by psychological, emotional, and social experiences during learning activities [26]. In contrast, perceived competence may be more closely associated with acquisition of specific knowledge and skills aligned with curricular objectives. According to this framework, learners may demonstrate improved competence while continuing to experience uncertainty, anxiety, or low self-efficacy, whereas others may report increased confidence despite relatively limited competence growth. Because ERS activities involve collaboration, communication, rapid problem-solving, and decision-making under time constraints, students may interpret and internalize the same learning experience differently based on prior experiences, emotional responses, personality characteristics, peer interactions, group dynamics, and perceived performance during the activity.

From an educational perspective, these findings highlight the importance of examining both competence and confidence outcomes, as reliance solely on competence measures may fail to identify students with low self-efficacy, overconfidence, or plateaued confidence development. The individual longitudinal plots also suggest that learners may respond differently to simulation-based educational experiences despite similar group-level trends.

The findings also have potential implications for CHN education within increasingly resource-constrained clinical environments. Nursing programs continue to experience challenges securing sufficient community-based placements, particularly opportunities involving underserved or medically complex populations. Simulation-based educational approaches such as ERS may provide an additional strategy to supplement limited clinical experiences by creating interactive learning environments that promote communication, environmental assessment, teamwork, prioritization, and clinical reasoning within community-focused scenarios. Although ERS should not be viewed as a replacement for direct patient care experiences, the present findings suggest that this approach may represent a feasible supplemental learning strategy that warrants further development and evaluation within CHN curricula.

Several limitations should be acknowledged. First, the study used a single-group pre–post design without a control or comparison group, which limits causal inference. Improvements observed over time may have been influenced by maturation, concurrent coursework, repeated testing, or other educational experiences rather than the ERS activity alone. Because the ERS activity was integrated within the required curriculum, withholding the educational experience from a subgroup of participants was not considered feasible or educationally appropriate. Therefore, findings should be interpreted cautiously as exploratory associations rather than definitive intervention effects.

Second, the study used a convenience sample from a single institution, which may limit generalizability to other nursing programs or educational settings. Third, competence was assessed using a self-reported dichotomous (Yes/No) checklist, which may have limited measurement sensitivity and contributed to ceiling effects observed at later measurement points. However, the checklist was intentionally designed to assess minimum safe competency expectations for undergraduate novice learners rather than expert-level mastery. This approach is conceptually consistent with competency-based nursing education and licensure frameworks emphasizing safe versus unsafe clinical practice. Given that undergraduate participants are early learners progressing from novice toward advanced beginner stages, dichotomous evaluation may provide clearer interpretation of foundational safe practice expectations than more complex mastery-oriented rating systems. Future studies should incorporate additional objective and performance-based assessment approaches capable of capturing more nuanced competency development.

Fourth, although the competence and confidence instruments demonstrated strong internal consistency within the current sample, comprehensive psychometric validation has not yet been established. Additional studies evaluating construct validity, criterion validity, and test–retest reliability are needed. Fifth, although demographic and experiential covariates did not appear to substantially influence longitudinal trajectories in sensitivity analyses, unmeasured factors such as academic ability, motivation, prior simulation exposure, or external clinical experiences may have contributed to variability in student responses.

Sixth, during the study, qualitative information was collected through structured debriefing sessions and facilitator reflections. Inclusion of qualitative findings could have provided additional insight into participants’ learning processes, emotional responses, and perceived challenges during the ERS activity. However, inclusion of both comprehensive quantitative and qualitative analyses within a single manuscript was beyond the scope and page limitations of the current article. Future publications will report the qualitative findings separately to provide a more in-depth understanding of participants’ learning experiences and perceptions of the ERS activity. Finally, outcomes were assessed only immediately after the ERS activity and again 10 weeks later, limiting evaluation of longer-term sustainability and translation into actual clinical performance.

## 5. Conclusions

There is limited evidence regarding the use of escape room simulation (ERS) in community health nursing (CHN) education. This study contributes to the literature by examining the feasibility and acceptability of ERS as a supplemental educational strategy within undergraduate CHN education. The findings suggest that ERS was associated with improvements in participants’ perceived competence and confidence while also providing engaging and interactive learning experiences related to home safety assessment, communication, clinical reasoning, teamwork, and community-based care. ERS may offer a feasible approach to partially address challenges associated with limited clinical placements by providing opportunities for participants to apply theoretical knowledge within realistic, low-risk simulated environments. On the other hand, even though ERS may provide valuable supplemental learning opportunities, hands-on, face-to-face clinical experiences remain essential and continue to be the most beneficial learning experiences for nursing participants.

Practical implications of the findings suggest that ERS may serve as a feasible alternative clinical practice strategy for both student education and healthcare staff training, particularly in situations where direct clinical exposure is limited, inconsistent, high-risk, or difficult to access. Future ERS design improvements may include enhanced faculty training, standardized facilitation guides, incorporation of interprofessional team-based scenarios, and integration of more diverse community-based situations such as domestic violence assessment, psychiatric crisis response, disaster preparedness, and other low-frequency but high-impact clinical events. Additional improvements may also include adaptive difficulty levels, structured reflective debriefing, objective performance-based assessment measures, and expanded longitudinal follow-up to better support skill retention, learner engagement, and transfer of learning into real-world clinical practice.

However, given the exploratory study design without a control group and the reliance on self-reported outcomes, the findings should be interpreted cautiously. Additional research using more rigorous controlled longitudinal designs, objective performance-based measures, qualitative evaluation, and larger multi-site samples is needed to better evaluate the educational impact and underlying mechanisms of ERS in CHN education and to support the development of evidence-based ERS approaches that enhance student preparedness for community-based nursing practice in settings where clinical placements remain limited.

## Figures and Tables

**Table 1:** Escape Room Simulation Setup.

Clue Box	Role and Responsibility	Learning Objectives
Living room	Assess home safety hazards and fall risks in the simulated environment.	Identify and evaluate environmental, physical, and psychological risks. Develop strategies for reporting and addressing safety concerns.
Kitchen	Evaluate food supply, nutrition, and access-to-care concerns	Assess the adequacy of food supplies and nutritional quality. Identify indicators of food insecurity. Recommend community resources and referrals.
Bedroom Bathroom	Review medication management practices and identify medication errors.	Detect and address medication errors. Implement proper medication management strategies. Develop plans for safe medication administration and patient education.

**Table 2:** Description of Participants Characteristics.

Variables	M ± *SD* or *N* (%)
Year of age	21.84 ± 2.795
Female	53 (94.6)
Race	
White	29 (51.8)
Black	11 (19.6)
Hispanic	7 (12.5)
Asian	7 (12.5)
Middle Eastern	1 (1.8)
Multi-race	1 (1.8)
Language	
English	41 (73.2)
Arabic	1 (1.8)
Mandarin	1 (1.8)
Vietnamese	1 (1.8)
Multilingual	12 (21.4)
Year of Education	16.14 ± 2.084
Full-time student	56 (100%)
Current work (weekly hr.)	10.88 ± 11.188
Pre-nursing work experience (yes)	46 (82.1)
Working places	3.78 ± 2.9
Acute care	19 (33.9)
Ambulatory clinics	4 (7.1)
Skilled nursing facilities	1 (1.8)
Rehab centers	1 (1.8)
Nursing home	4 (7.1)
Assisted living	4 (7.1)
Home health	4 (7.1)
Multi-site	8 (14.3)
Length of employment (months)	41.22 ± 24.235
Previous escape room experience (Yes)	13 (23.2)
Previous escape room experience (hrs.)	2.77 ± 2.713

**Table 3:** Descriptive statistics for student’s competence, confidence and escape room experience.

	Min	Median	Mean	Max	SD	Effect Sizes (Cohen’s d)
CHN competence						
Pre-competence (T1)	4.00	16.50	14.98	18.00	3.88	-
Post-competence (T2)	13.00	18.00	17.39	18.00	1.27	d = 0.810 (T2 vs. T1)
10 week-competence (T3)	14.00	18.00	17.85	18.00	0.62	d = 0.799 (T3 vs. T1)
CHN confidence						
Pre-confidence (T1)	450	1315	1295.56	1700	276.08	-
Post-confidence (T2)	1040	1545	1535.98	1800	204.09	d = 1.241 (T2 vs. T1)
10 week-confidence (T3)	1380	1720	1689.45	1800	111.26	d = 1.494 (T3 vs. T1)
Perceived ERS experience						
Escape room experience perception scale (T2)	13.00	63.00	59.96	65	8.19	-

**Table 4:** Regression analysis for competence.

	Coefficients	Stand Error	*p*-Value	95% CI
Intercept (pre-competency, T_1_)	15	0.319	<0.001	(14.4, 15.6)
Post-competency T_2_—pre-competency T_1_	2	0.376	<0.001	(1.7, 3.1)
T_3_-competency—pre-competency T_1_	3	0.378	<0.001	(2.1, 3.6)

**Table 5:** Regression analysis for confidence.

	Coefficients	Stand Error	*p*-Value	95% CI
Intercept (pre-competency, T_1_)	1291	29.0	<0.001	(1234, 1347)
Post-competency T_2_—pre-competency T_1_	245	30.1	<0.001	(186, 304)
T_3_-competency—pre-competency T_1_	403	31.8	<0.001	(341, 466)

## Data Availability

The datasets generated and analyzed during the current study are not publicly available because the study involved undergraduate nursing participants, a population considered vulnerable in educational research, and public release may risk participant confidentiality. However, de-identified data may be made available from the corresponding author upon reasonable request, contingent upon institutional review board (IRB) approval and completion of applicable data use and confidentiality agreements.

## Supplementary Materials

The following supporting information can be downloaded at: https://www.mdpi.com/article/10.3390/nursrep16060189/s1, The supplementary files include the study protocol, simulation procedures and guides, standardized patient materials, assessment tools, scoring instruments, debriefing materials, and data collection instruments used in the study. References [27,28,29,30,31,32,33,34,35,36,37,38,39,40,41,42,43] are cited in SM.

## Public Involvement Statement

No public involvement in any aspect of this research.

## Guidelines and Standards Statement

This manuscript was drafted in accordance with the Transparent Reporting of Evaluations with Nonrandomized Designs (TREND) Statement for nonrandomized intervention studies.

## Abbreviations

The following abbreviations are used in this manuscript:

ERS	Escape Room Simulation
CHN	Community Health Nursing
QCC	Quad Council Coalition
SDOH	Social Determinants of Health
