# Supplementary material for: Feasibility of Escape Room Simulation in Community Health Nursing Education: A Quasi-Experimental Study of Student Perceptions of Competence and Confidence
Source: Nurs Rep. 2026 May 29;16(6):189. doi: 10.3390/nursrep16060189 (PMC13305034; doi:10.3390/nursrep16060189)
Supplement: Supplementary file 1 [file nursrep-16-00189-s001.zip › nursrep-4286939-supplementary.pdf]

Supplementary File SI
Study Protocol: Escape Room Simulation in Community Health Nursing Education

1. IRB Approval and Study Preparation

Before data collection begins, the research team will obtain approval from the university Institutional Review Board. All study materials, including the consent form, recruitment script, survey instruments, data collection forms, and study procedures, will be submitted for review. Faculty and student workers assisting with the study will complete required human subjects protection training.

2. Participant Recruitment

Eligible participants will include undergraduate nursing students enrolled in the Community Health Nursing course. Students will be informed that the escape room simulation is part of the course learning experience, but participation in the research data collection is voluntary. Students will be told that choosing not to participate, or withdrawing from the study, will not affect their course grade, clinical evaluation, or relationship with faculty.

3. Informed Consent

Before completing any research surveys, students will receive an informed consent form explaining the study purpose, procedures, risks, benefits, confidentiality protections, and participant rights. Students who agree to participate will provide consent before completing the baseline questionnaires. Students may stop participation at any time without penalty.

4. Baseline Data Collection: Pre-Simulation

At the beginning of the escape room activity, participating students will complete baseline measures before entering the simulation. These include the Community Health Nursing competency checklist and the Community Health Nursing confidence scale.

5. Escape Room Simulation

Students will participate in the escape room simulation during scheduled clinical hours at the School of Nursing Simulation Center. Students will work in groups to complete community health nursing case-based challenges. The simulation includes home assessment, identification of social determinants of health, patient safety concerns, problem solving, teamwork, communication, and clinical reasoning activities.

The entire study activity will require approximately 1.5–2 hours to complete, with an estimated duration of 90–105 minutes from beginning to end, depending on transition timing and the depth of discussion during the structured debriefing session. The estimated time breakdown is as follows:

- Student arrival and orientation: 15 minutes
- Escape room instructions/scenario review: 5 minutes
- Escape room activity and transition: 25 minutes
- Final structured debriefing: 45 minutes

The simulation sequence was designed to reflect key components of community-based nursing care, beginning with environmental assessment and patient intake, followed by identification of social determinants of health, collaborative problem solving, and faculty-guided reflective debriefing.

6. Immediate Post-Simulation Data Collection

Immediately after the simulation, students will complete the CHN competency checklist and CHN confidence scale again. Students will also complete the Escape Room Experience Perception Scale to assess their perceptions of enjoyment, feasibility, usefulness, relevance, and perceived outcomes of the activity.

7. Debriefing

After the escape room activity, faculty will facilitate a structured group debriefing. Students will reflect on their experience, clinical decision-making, teamwork, communication, and application of CHN concepts. Feedback from students and instructors may be summarized using descriptive thematic analysis.

8. Follow-Up Data Collection

Approximately 10 weeks after the simulation, students will complete the CHN competency checklist and CHN confidence scale for a third time. This follow-up assessment will examine whether perceived competence and confidence are maintained over time.

9. Data Management and Confidentiality

All data will be stored securely. Student responses will be de-identified before analysis. Research data will not be used for course grading. Only approved research team members will have access to the study data.

Supplementary File SII
Data Collection Procedure for Escape Room Simulation in Community Health Nursing Education

The following table outlines the key components of the study, including the purpose of each session, faculty roles, estimated duration, and timing of data collection throughout the study process.

Session Component	Purpose	Data Collected
Welcome, orientation, and informed consent (10–15 min)	Review study procedures and simulation expectations. (Facilitator)	Consent
Pre-simulation scenario introduction and chart review (10–15 min)	Introduce community health case scenario and orient students to simulation activities. (Facilitator)	• CHN Competency Checklist (baseline)<br>• CHN Confidence Scale (baseline)
Escape room simulation activity (40–45 min)	Simulate community health nursing assessment, teamwork, environmental safety evaluation, and clinical reasoning. (Facilitator)	
Group debriefing session (45 min)	Facilitate reflective learning, communication, teamwork discussion, and integration of CHN concepts. (Facilitator)	• CHN Competency Checklist (post)<br>• CHN Confidence Scale (post)<br>• Escape Room Experience Perception Scale (EREPS)
Follow-up assessment (10 weeks post-simulation)	Evaluate maintenance of perceived competence and confidence over time.	• CHN Competency Checklist (follow-up)<br>• CHN Confidence Scale (follow-up)

Supplementary File SIII
Escape Room Simulation Guide for Community Health Nursing Care

Guide for Students

Simulation Overview

Welcome to the Community Health Simulation Escape Room!

- Today you will participate in an evolving community health nursing simulation experience designed to help you apply what you have learned in real-world community settings.
- You will participate in groups of four students and go through 2 stations (see detail below). Your team's goal is to complete a comprehensive home assessment and patient intake during a simulated home visit while working together to "escape" the room.
- During the process, you will become familiar with the simulated apartment environment, assessment equipment, and patient care challenges commonly encountered in community health nursing practice.
- You will have 40–45 minutes to complete the escape room activities.
- Remember your team cannot successfully "escape" without completing all required tasks and assessments.

What Makes This Simulation Different?

- This is more than just an escape room. The experience has evolved into a multi-modal community health nursing simulation that combines:
    - Home assessment
    - Standardized patient (SP) interaction
    - Social determinants of health (SDoH) screening
    - Collaborative team care planning
    - Faculty-guided debriefing and reflection
- The simulation is designed to follow the flow of real community-based nursing care from assessing the home environment, to communicating with patients, to developing interdisciplinary care plans and patient education strategies.
- Throughout the experience, focus on teamwork, communication, critical thinking, prioritization, and identifying both medical and social needs that may affect patient health and well-being.

Student Arrival and Orientation

- Students are required to arrive 15 minutes early.
- Faculty will provide:
    - Escape room scenario and directions
    - Simulation instructions
    - Care planning instructions
    - Directions for all simulation stations
- Faculty will review the expectations, workflow, and transition procedures with students before entering the simulation activities.

Escape Room Simulation Rules

- No cell phones or tablets can be brought into the room.
- You may receive hints from the observing faculty member. One minute will be added to your time for each hint.
- One minute will be added to your time for each task that was missed.
- You will be notified when you have 10 minutes remaining.
- All assessment cues can be found in each room.
- Examine the entire room.
- Please do not force anything open. If you try to force things, you will break them.

- Try to complete tasks in order.
- Do not unplug any equipment.
- Do not pull anything off the wall.
- Do not do anything to the manikin that you would not do to a person.
- Do not move the manikin from where he/she is.
- Once you 'escape', a faculty member will come to the room to go over any equipment, if you have questions.
- Please do not discuss the details of this activity outside of the debrief!

Station 1: Standardized Patient (SP) Interaction

- **Purpose**
  - Simulate patient-centered communication during a community health nursing home visit.
  - Promote therapeutic communication, interviewing skills, rapport building, and assessment of psychosocial and social determinants of health (SDoH) concerns.
- **Activities**
  - A standardized participant is present within the apartment simulation to create a realistic home visit encounter.
  - Students introduce themselves, establish rapport, and explain the purpose of the home health visit.
  - Students ask assessment questions related to:
    - Current health concerns
    - Caregiver responsibilities
    - Daily functioning
    - Medication management
    - Financial concerns
    - Social support
    - Transportation barriers
    - Psychosocial stressors
  - Students demonstrate therapeutic communication and patient-centered interviewing techniques throughout the encounter.
  - Students collaboratively complete SDoH assessment and screening activities while interacting with the patient.

Station 2: Home Assessment

- **Purpose**
  - Simulate a comprehensive community-based home safety and environmental assessment.
  - Promote teamwork, communication, environmental assessment, clinical reasoning, prioritization, and problem-solving skills.
- **Activities**
  - Students conduct a comprehensive home assessment by systematically evaluating the:
    - Living room
    - Kitchen
    - Bedroom
    - Bathroom
  - Students identify environmental safety concerns, medication-related risks, patient assessment cues, and household hazards embedded throughout the apartment setting.
  - Students assess for:
    - Fall risks
    - Medication safety concerns
    - Food insecurity
    - Accessibility barriers

        - Clutter and sanitation concerns
        - Bathroom safety risks
        - Chronic disease management concerns

Structured and Group Debriefing

- **Purpose**
    - Facilitate reflective learning and integration of clinical experiences across all simulation phases.
- **Debriefing Focus**
    - Student reactions and reflections
    - Clinical decision-making processes
    - Team communication and collaboration
    - SDoH considerations
    - Community-based nursing interventions
    - Lessons learned from the evolving simulation process

Guide for Faculty Facilitator

Faculty facilitators follow standardized moderator guides developed in alignment with the International Nursing Association for Clinical Simulation and Learning (INACSL) Healthcare Simulation Standards of Best Practice™ to ensure consistency across student groups.

- Prior to the simulation, faculty conduct a structured pre-briefing that includes:
    - Review of learning objectives and expected student roles
    - Orientation to the apartment-style simulation environment, standardized patient interaction procedures, and environmental clue identification processes.
    - Explanation of ERS rules, timing, and team expectations
    - Introduction to available supplies, assessment tools, and documentation materials
    - Establishment of psychological safety, confidentiality, and respectful communication expectations
- During the simulation, faculty primarily serve as observers rather than active participants. Faculty monitor and document student performance related to:
    - Therapeutic communication and interviewing skills
    - Use of the SDOH screening tool to assess health and socioeconomic stressors
    - Cultural sensitivity and patient-centered communication
    - Clinical reasoning and prioritization
    - Home safety and environmental assessment
    - Teamwork, collaboration, and delegation
    - Identification of patient care concerns and social risks
    - Problem-solving and decision-making processes
- Faculty intentionally minimize direct instruction during the ERS activity to preserve simulation realism, learner autonomy, and active problem-solving.
- Faculty do not directly provide answers or guide students toward solutions unless:
    - A major patient safety concern occurs
    - Students demonstrate unsafe behaviors
    - Significant communication breakdown disrupts the activity
    - Technical or operational issues interfere with simulation progression
- Faculty monitor students' emotional and psychological safety throughout the activity and maintain a supportive, nonjudgmental learning environment.
- Following completion of the ERS activity, faculty facilitate a structured reflective debriefing session focused on:
    - Clinical reasoning and prioritization
    - Team communication and collaboration

    - Therapeutic communication with the standardized patient
    - Identification of SDOH-related risks and community resources
    - Home safety assessment findings
    - Student reflections, emotional responses, and lessons learned
    - Application of simulation experiences to real-world community health nursing practice
- Faculty is responsible to reset the simulation apartment between student groups, and ensure the simulation environment remains standardized across student experiences.

Supplementary File SIV
Station 1 Standardized Patient (SP) Interaction for Community Health Nursing Care Escape Room Simulation
Standardized Patient Scripts

Screening Tool:
Students will use the Accountable Health Communities Health-Related Social Needs Screening Tool to guide their interview with the standardized patient. They are encouraged to ask for the same information in more of a conversational manner. The text in blue indicates the standardized patient script.

Living Situation

1. What is your living situation today?
□ I have a steady place to live
□ I have a place to live today, but I am worried about losing it in the future
□ I do not have a steady place to live (I am temporarily staying with others, in a hotel, in a shelter, living outside on the street, on a beach, in a car, abandoned building, bus or train station, or in a park)

Standardized Patient Scripts
"I'd say I'm a little worried (looks down or away then back again). I mean, we're ok now. But, sometimes Mr. Lopez's medical bills get to be a lot. So, I guess we could maybe not have enough money sometime in the future. You know? And also, the landlord could even raise our rent, that's happened to people, I've heard about it."

2. Think about the place you live. Do you have problems with any of the following?
CHOOSE ALL THAT APPLY
□ Pests such as bugs, ants, or mice
□ Mold
□ Lead paint or pipes
□ Lack of heat
□ Oven or stove not working
□ Smoke detectors missing or not working
□ Water leaks
□ None of the above

Standardized Patient Scripts
Pests- "Yes, sometimes we have bugs in our home. I do my best to keep things clean. It's frustrating to clean and then to see bugs. It makes me embarrassed. (looks away, shakes head)"

Mold- "We did have mold in the bathroom, but the landlord sent someone in to clean it up and fix up the bathroom. So that seems better now."

Lead paint- "I don't think we have that. How would I know?"

Lack of heat- "Our heat works fine. But I keep it low in the winter…or we'll get a really big bill."

Oven or stove- "We don't have an oven. When we moved in, we got to choose between having an oven or a washer/dryer in our apartment. I can cook good enough using the microwave and an electric burner my daughter gave us. So, I picked having the washer/dryer because it's hard to go to the basement to do that. Plus, back then we used to get Meals on Wheels…we don't have that anymore (shakes head). We thought our plan

was good…but then the washer/dryer broke so now I can't use it and I have to go down to the basement of the building which isn't easy for me (shakes head)."

Smoke detectors- "We have those but they've never gone off. I assume they work fine, but goodness (eyes wide), if there was a fire, it'd be real hard to get Mr. Lopez out of the apartment and out of the building! The elevator wouldn't work if there's a fire and he's just not steady enough for me to get him down the stairs. I don't know what we'd do!"

Water leaks- "I haven't noticed any water leaks."

Food

Some people have made the following statements about their food situation. Please answer whether the statements were OFTEN, SOMETIMES, or NEVER true for you and your household in the last 12 months.
3. Within the past 12 months, you worried that your food would run out before you got money to buy more.

□ Often true
□ Sometimes true
□ Never true

Standardized Patient Scripts
"I guess I'd say my answer is often true. But, you know, we make sure not to buy expensive stuff. I shop the sales and we eat a lot of boxed foods because they can stay good in the cabinets for longer. It's hard for me to get to the store sometimes. I get tired and I just don't like leaving Mr. Lopez home alone for too long. So, I don't go to the store a whole lot. We have enough food to get by most of the time, it's just maybe not the best food. Sometimes we just ignore the expiration date and eat it anyway. It's really hard (looks down). Sometimes Mr. Lopez says he's still hungry, and that makes me feel sad. We o manage to get by, I think. I really try to do the best I can."

4. Within the past 12 months, the food you bought just didn't last and you didn't have money to get more.

□ Often true
□ Sometimes true
□ Never true

Standardized Patient Scripts
"As I said, I buy boxed food so it can last pretty long and sometimes I ignore the expiration date. We don't do a lot of fresh foods like vegetables or meat. Because that stuff's expensive, and it's also hard for me to get out to the store to get it. We've never run completely out of something to east, but we have gotten close a couple of times."

Transportation

5. In the past 12 months, has lack of reliable transportation kept you from medical appointments, meetings, work or from getting things needed for daily living?

□ Yes
□ No

Standardized Patient Scripts
"We do have a car; the same one we've had for a long time now. I love my good old car, but, it's getting to that age where it's breaking down a lot. Cars just cost so much money to fix nowadays. We don't always have the money to fix it right away, so I can't always depend on having it to use. Plus, gas prices have gotten so high! I

am lucky to have a neighbor who can give me a ride if my car isn't working or I can't afford to put gas in it. She does go out of town to visit her grandkids a lot, so it's not all the time that she can drive me…but she's so nice to help when she is here. There is also a bus route near here, but I couldn't get Mr. Lopez on a bus."

Utilities

6. In the past 12 months has the electric, gas, oil, or water company threatened to shut off services in your home?

□ Yes
□ No
□ Already shut off

Standardized Patient Scripts
"No, we haven't had any of that shut off…thank goodness. I always pay our bills before anything else. We get by. Like I said, we turn the heat low in the winter and don't really use the air conditioner in the summer."

Safety

Because violence and abuse happens to a lot of people and affects their health we are asking the following questions.

7. How often does anyone, including family and friends, physically hurt you?

□ Never (1)
□ Rarely (2)
□ Sometimes (3)
□ Fairly often (4)
□ Frequently (5)

Standardized Patient Scripts
"That has never happened to us."

8. How often does anyone, including family and friends, insult or talk down to you?

□ Never (1)
□ Rarely (2)
□ Sometimes (3)
□ Fairly often (4)
□ Frequently (5)

Standardized Patient Scripts
"Mr. Lopez and I get along just fine. We don't really talk to many other people, so that has neve happened to us."

9. How often does anyone, including family and friends, threaten you with harm?

□ Never (1)
□ Rarely (2)
□ Sometimes (3)
□ Fairly often (4)
□ Frequently (5)

Standardized Patient Scripts
"That'd sure be awful. I'm glad that has never happened to us."

10. How often does anyone, including family and friends, scream or curse at you?
□ Never (1)
□ Rarely (2)
□ Sometimes (3)
□ Fairly often (4)
□ Frequently (5)

Standardized Patient Scripts
"We don't use bad language and even if I screamed, Mr. Lopez couldn't hear me. We get along good with each other and anyone we happen to meet, so that never happens to us."

Financial Strain

11. How hard is it for you to pay for the very basics like food, housing, medical care, and heating? Would you say it is:
□ Very hard
□ Somewhat hard
□ Not hard at all

Standardized Patient Scripts
"Money is tight, so guess I'd say very hard. We get social security, but it sure goes fast with rent, bills, and Mr. Lopez's medical bills and prescriptions. We do have a small savings account that we need to use from time to time. We used most of our savings after Mr. Lopez had his stroke. Right now, I guess we're okay. There's surely people worse off than we are. But, I do worry about it a lot (looks down or away), and sometimes I can't sleep for worrying."

Employment

12. Do you want help finding or keeping work or a job?
□ Yes, help finding work
□ Yes, help keeping work
□ I do not need or want help

Standardized Patient Scripts
Laughs. "No, no more work for me. It's a big enough job to take care of my husband. And now with this cancer, I couldn't work if I wanted to."

Family and Community Support

13. If for any reason you need help with day-to-day activities such as bathing, preparing meals, shopping, managing finances, etc., do you get the help you need?
□ I don't need any help
□ I get all the help I need
□ I could use a little more help
□ I need a lot more help

Standardized Patient Scripts
"I'd say I could use a little more help. Definitely now because I'm tired and run down with this cancer I have in my chest. It's all just a lot..." (Looks away.) "I do my best. We get by. But, it's not always easy to take care of the both of us. You know, Mr. Lopez used to pay the bills and stuff, but his eyes aren't great anymore. It's been a lot for me to learn. Cooking and stuff I don't mind doing. Laundry is harder because since our washer/dryer broke

I have to carry everything down to the basement and back. It's heavy for me to carry, and hard to get down there and back again. I don't know what he'll do if I die before him."

14. How often do you feel lonely or isolated from those around you?
□ Never
□ Rarely
□ Sometimes
□ Often
□ Always

Standardized Patient Scripts
"I guess...I guess sometimes it feels lonely. I do always have Mr. Lopez. We talk and listen to the radio. He's a great partner. But, it'd be nice to get out once in a while, talk to other people too. I wish we could still go to Mass at the church. And, I do wish we got to see our daughters and grandchildren more often. I miss them. They all live out of state and have their own lives. We miss watching the news on TV, but ours broke so we just have the radio now. It's good, but might be nice to see other people – even if it was TV! We do have out little cat, Fluffy, and a dog, Max, that our daughter brought us."

Education

15. Do you speak a language other than English at home?
□ Yes
□ No

Standardized Patient Scripts
"No."

16. Do you want help with school or training? For example, starting or completing job training or getting a high school diploma, GED or equivalent.
□ Yes
□ No

Standardized Patient Scripts
"I think I'm past that time in my life!" Laughs.

Physical Activity

17. In the last 30 days, other than the activities you did for work, on average, how many days per week did you engage in moderate exercise (like walking fast, running, jogging, dancing, swimming, biking, or other similar activities)?
□ 0
□ 1
□ 2
□ 3
□ 4
□ 5
□ 6
□ 7

Standardized Patient Scripts

“Moderate exercise isn't really what I would call it. I do like to walk. Our dog, Max, likes to walk. He’s a good little boy. I try to take him on a walk outside every day to do his business. He’s trained to go on the little pads if we can’t take our walk. I haven’t been able to go lately because I’m so tired. But normally, I do walk a little. It's good for my bones they say. But, again, I don't like to leave Mr. Lopez alone for too, too long."

18. On average, how many minutes did you usually spend exercising at this level on one of those days?
□ 0
□ 10
□ 20
□ 30
□ 40
□ 50
□ 60
□ 90
□ 120
□ 150 or greater

Standardized Patient Scripts
"Just a short walk is what I do, when I'm able."

Substance Use

The next questions relate to your experience with alcohol, cigarettes, and other drugs. Some of the substances are prescribed by a doctor (like pain medications), but only count those if you have taken them for reasons or in doses other than prescribed. One question is about illicit or illegal drug use, but we only ask in order to identify community services that may be available to help you.

19. How many times in the past 12 months have you had 5 or more drinks in a day (males) or 4 or more drinks in a day (females)? One drink is 12 ounces of beer, 5 ounces of wine, or 1.5 ounces of 80-proof spirits.

☐ Never

☐ Once or Twice

☐ Monthly

☐ Weekly

☐ Daily or Almost Daily

Standardized Patient Scripts
"We don't have drinks too often. Just for special occasions, or when the girls come to visit, I'd say. I'm fearful of falling and certainly don't want Mr. Lopez to fall."

20. How many times in the past 12 months have you used tobacco products (like cigarettes, cigars, snuff, chew, electronic cigarettes)?

☐ Never

☐ Once or Twice

☐ Monthly

☐ Weekly

☐ Daily or Almost Daily

Standardized Patient Scripts
"We don't smoke. When we were much younger, we did. But no smoking for us in years."

21. How many times in the past year have you used prescription drugs for non-medical reasons?

☐ Never

☐ Once or Twice

☐ Monthly

☐ Weekly

☐ Daily or Almost Daily

Standardized Patient Scripts
"Do you mean take medicine that's not from my doctor?" "We're definitely not taking any medicines that we aren't supposed to."

22. How many times in the past year have you used illegal drugs?

☐ Never

☐ Once or Twice

☐ Monthly

☐ Weekly

☐ Daily or Almost Daily

Standardized Patient Scripts
"Oh we have never done that."

Mental Health

23. Over the past 2 weeks, how often have you been bothered by any of the following problems?

a. Little interest or pleasure in doing things?
□ Not at all (0)
□ Several days (1)
□ More than half the days (2)
□ Nearly every day (3)

Standardized Patient Scripts
"I guess I would say several days. Though even more lately because I've been sick and I just don't have energy like I usually do. It'd be nice to get out and do stuff again, not always feel like we're stuck in our house."

b. Feeling down, depressed, or hopeless?

□ Not at all (0)
□ Several days (1)
□ More than half the days (2)
□ Nearly every day (3)

Standardized Patient Scripts

"Umm, I guess several days is how I'd answer again for this one. It's tough being home, tougher when you're not feeling good. It's hard to know that we don't have everything we need...like food that's good for my husband's diabetes and stuff. That frustrates me. I didn't think that would happen to us (shakes head). I just didn't see us living this way, stuck at home, not seeing our daughters much. It's really hard sometimes."

24. Stress means a situation in which a person feels tense, restless, nervous, or anxious, or is unable to sleep at night because his or her mind is troubled all the time. Do you feel this kind of stress these days?
□ Not at all
□ A little bit
□ Somewhat
□ Quite a bit
□ Very much

Standardized Patient Scripts
"I think that's how I feel. Yes, definitely, sometimes. When I get scared about being able to get us the stuff we need, being able to do it all myself. It's hard you know and it keeps me awake at night."

Disabilities

25. Because of a physical, mental, or emotional condition, do you have serious difficulty concentrating, remembering, or making decisions?
□ Yes
□ No

Standardized Patient Scripts
"No, not me. Mr. Lopez sometimes, but that's why I'm here to help him!"

26. Because of a physical, mental, or emotional condition, do you have difficulty doing errands alone such as visiting a doctor's office or shopping?
□ Yes
□ No

Standardized Patient Scripts
"We get to our appointments. Sometimes it's a lot of arranging, especially if the car isn't working. But I make sure to get us there. Though, I do like the visits like this on the computer. Much easier. I am using my neighbor's computer right now, we don't have one and we don't pay for the internet either, just another bill to worry about. But, if we could figure out a way, visits like this would be really helpful, especially where they're really just talking. It makes it a lot easier for us."

CENTERS FOR MEDICARE & MEDICAID SERVICES

The Accountable Health Communities Health-Related Social Needs Screening Tool

What's the Accountable Health Communities (AHC) Health-Related Social Needs (HRSN) Screening Tool?

We at the Centers for Medicare & Medicaid Services (CMS) Center for Medicare and Medicaid Innovation (CMMI) made the Accountable Health Communities (AHC) Health-Related Social Needs (HRSN) Screening Tool to use in the AHC Model.[1] We're testing to see if systematically finding and dealing with the health-related social needs of Medicare and Medicaid beneficiaries has any effect on their total health care costs and makes their health outcomes better.

Why is the AHC HRSN Screening Tool important?

Growing evidence shows that if we deal with unmet HRSNs like homelessness, hunger, and exposure to violence, we can help undo their harm to health. Just like with clinical assessment tools, providers can use the results from the HRSN Screening Tool to inform patients' treatment plans and make referrals to community services.

What does the AHC HRSN Screening Tool mean for me?

Screening for HRSNs isn't standard clinical practice yet. We're making the AHC HRSN Screening Tool a standard screening across all the communities in the AHC Model. We're sharing the AHC HRSN Screening Tool for awareness.

What's in the AHC HRSN Screening Tool?

In a National Academy of Medicine discussion paper,[2] we shared the 10-item HRSN Screening Tool. The Tool can help providers find out patients' needs in these 5 core domains that community services can help with:

- Housing instability
- Food insecurity
- Transportation problems
- Utility help needs

[1] United States, U.S. Department of Health and Human Services, Centers for Medicare & Medicaid Services. (2017, September 05). Accountable Health Communities Model. https://innovation.cms.gov/initiatives/ahcm.
[2] Billioux, A., MD, DPhil, Verlander, K., MPH, Anthony, S., DrPH, & Alley, D., PhD. (2017). Standardized Screening for Health-Related Social Needs in Clinical Settings: The Accountable Health Communities Screening Tool. National Academy of Medicine Perspectives, 1-9. https://nam.edu/wp-content/uploads/2017/05/Standardized-Screening-for-Health-Related-Social-Needs-in-Clinical-Settings.pdf.

- Interpersonal safety

In the final version below, we made small revisions to the original 10 questions based on cognitive testing we did since we shared the first version. In the final version we also included questions in 8 supplemental domains that we haven't shared before:

- Financial strain
- Employment
- Family and community support
- Education
- Physical activity
- Substance use
- Mental health
- Disabilities

Who should use the AHC HRSN Screening Tool?

The questions in the AHC HRSN Screening Tool are meant to be used for individual respondents who answer the questions themselves. A parent or caregiver can answer for an individual, too, if that makes more sense. Clinicians and their staff can easily use this short tool as part of their busy clinical workflows with people of all different ages, backgrounds, and settings.

In the next 5 years, hundreds of participating clinical delivery sites across the 32 AHCs will screen over 7 million Medicare and Medicaid beneficiaries using the 10 core domain questions. The AHCs can also choose to add any of the supplemental domain questions into their standard screening processes.

Who made the AHC HRSN Screening Tool?

We made this tool with a panel of experts from around the country including:

- Tool developers
- Public health and clinical researchers
- Clinicians
- Population health and health systems executives
- Community-based organization leaders
- Federal partners

We got permission from the original authors of the questions to use, copy, modify, publish, and distribute the questions for the AHC Model and our use only. Based on feedback from the original question authors, CMS has created this table to specify the citation and notification process for each screening question in the AHC HRSN Screening Tool if the questions are used outside of CMS and the AHC Model.

AHC HRSN Screening Tool Core Questions

If someone chooses the underlined answers, they might have an unmet health-related social need.

Living Situation

1. **What is your living situation today?**[3]
   - ☐ I have a steady place to live
   - ☐ I have a place to live today, but **I am worried** about losing it in the future
   - ☐ I do not have a steady place to live (I am temporarily staying with others, in a hotel, in a shelter, living outside on the street, on a beach, in a car, abandoned building, bus or train station, or in a park)

2. **Think about the place you live. Do you have problems with any of the following?**[4]
   CHOOSE ALL THAT APPLY
   - ☐ Pests such as bugs, ants, or mice
   - ☐ Mold
   - ☐ Lead paint or pipes
   - ☐ Lack of heat
   - ☐ Oven or stove not working
   - ☐ Smoke detectors missing or not working
   - ☐ Water leaks
   - ☐ None of the above

Food

Some people have made the following statements about their food situation. Please answer whether the statements were OFTEN, SOMETIMES, or NEVER true for you and your household in the last 12 months. [5]

3. **Within the past 12 months, you worried that your food would run out before you got money to buy more.**
   - ☐ Often true
   - ☐ Sometimes true
   - ☐ Never true

---

[3] National Association of Community Health Centers and partners, National Association of Community Health Centers, Association of Asian Pacific Community Health Organizations, Association OPC, Institute for Alternative Futures. (2017). PRAPARE. http://www.nachc.org/research-and-data/prapare/

[4] Nuruzzaman, N., Broadwin, M., Kourouma, K., & Olson, D. P. (2015). Making the Social Determinants of Health a Routine Part of Medical Care. Journal of Healthcare for the Poor and Underserved, 26(2), 321-327.

[5] Hager, E. R., Quigg, A. M., Black, M. M., Coleman, S. M., Heeren, T., Rose-Jacobs, R., Frank, D. A. (2010). Development and Validity of a 2-Item Screen to Identify Families at Risk for Food Insecurity. Pediatrics, 126(1), 26-32. doi:10.1542/peds.2009-3146

4. **Within the past 12 months, the food you bought just didn't last and you didn't have money to get more.**
   - ☐ Often true
   - ☐ Sometimes true
   - ☐ Never true

Transportation

5. **In the past 12 months, has lack of reliable transportation kept you from medical appointments, meetings, work or from getting things needed for daily living?[6]**
   - ☐ Yes
   - ☐ No

Utilities

6. **In the past 12 months has the electric, gas, oil, or water company threatened to shut off services in your home?[7]**
   - ☐ Yes
   - ☐ No
   - ☐ Already shut off

Safety

Because violence and abuse happens to a lot of people and affects their health we are asking the following questions. [8]

7. **How often does anyone, including family and friends, physically hurt you?**
   - ☐ Never (1)
   - ☐ Rarely (2)
   - ☐ Sometimes (3)
   - ☐ Fairly often (4)
   - ☐ Frequently (5)

---

[6] National Association of Community Health Centers and Partners, National Association of Community Health Centers, Association of Asian Pacific Community Health Organizations, Association OPC, Institute for Alternative Futures. (2017). PRAPARE. http://www.nachc.org/research-and-data/prapare/

[7] Cook, J. T., Frank, D. A., Casey, P. H., Rose-Jacobs, R., Black, M. M., Chilton, M., . . . Cutts, D. B. (2008). A Brief Indicator of Household Energy Security: Associations with Food Security, Child Health, and Child Development in US Infants and Toddlers. Pediatrics, 122(4), 867-875. doi:10.1542/peds.2008-0286

[8] Sherin, K. M., Sinacore, J. M., Li, X. Q., Zitter, R. E., & Shakil, A. (1998). HITS: a Short Domestic Violence Screening Tool for Use in a Family Practice Setting. Family Medicine, 30(7), 508-512

8. How often does anyone, including family and friends, insult or talk down to you?

- ☐ Never (1)
- ☐ Rarely (2)
- ☐ Sometimes (3)
- ☐ Fairly often (4)
- ☐ Frequently (5)

9. How often does anyone, including family and friends, threaten you with harm?

- ☐ Never (1)
- ☐ Rarely (2)
- ☐ Sometimes (3)
- ☐ Fairly often (4)
- ☐ Frequently (5)

10. How often does anyone, including family and friends, scream or curse at you?

- ☐ Never (1)
- ☐ Rarely (2)
- ☐ Sometimes (3)
- ☐ Fairly often (4)
- ☐ Frequently (5)

A score of 11 or more when the numerical values for answers to questions 7-10 are added shows that the person might not be safe.

AHC HRSN Screening Tool Supplemental Questions

Financial Strain

11. How hard is it for you to pay for the very basics like food, housing, medical care, and heating? Would you say it is:[9,]

- ☐ Very hard
- ☐ Somewhat hard
- ☐ Not hard at all

Employment

12. Do you want help finding or keeping work or a job?[10]

- ☐ Yes, help finding work
- ☐ Yes, help keeping work
- ☐ I do not need or want help

Family and Community Support

13. If for any reason you need help with day-to-day activities such as bathing, preparing meals, shopping, managing finances, etc., do you get the help you need?[11]

- ☐ I don't need any help
- ☐ I get all the help I need
- ☐ I could use a little more help
- ☐ I need a lot more help

14. How often do you feel lonely or isolated from those around you?[12]

- ☐ Never
- ☐ Rarely
- ☐ Sometimes
- ☐ Often
- ☐ Always

---

9 Hall, M. H., Matthews, K. A., Kravitz, H. M., Gold, E. B., Buysse, D. J., Bromberger, J. T., . . . Sowers, M. (2009). Race and Financial Strain are Independent Correlates of Sleep in Midlife Women: The SWAN Sleep Study. Sleep, 32(1), 73-82. doi:10.5665/sleep/32.1.73

10 Identifying and Recommending Screening Questions for the Accountable Health Communities Model (2016, July) Technical Expert Panel discussion conducted at the U.S. Department of Health and Human Services, Centers for Medicare & Medicaid Services, Baltimore, MD.

11 Kaiser Permanente. (2012, June). Medicare Total Health Assessment Questionnaire. Retrieved from https://mydoctor.kaiserpermanente.org/ncal/Images/Medicare%20Total%20Health%20Assessment%20Questionnaire_tcm75-487922.pdf

12 Anderson, G. Oscar and Colette E. Thayer. Loneliness and Social Connections: A National Survey of Adults 45 and Older. Washington, DC: AARP Research, September 2018. https://doi.org/10.26419/res.00246.001

Education

15. Do you speak a language other than English at home?[13]

- ☐ Yes
- ☐ No

16. Do you want help with school or training? For example, starting or completing job training or getting a high school diploma, GED or equivalent.[14]

- ☐ Yes
- ☐ No

Physical Activity

17. In the last 30 days, other than the activities you did for work, on average, how many days per week did you engage in moderate exercise (like walking fast, running, jogging, dancing, swimming, biking, or other similar activities)?[15]

- ☐ 0
- ☐ 1
- ☐ 2
- ☐ 3
- ☐ 4
- ☐ 5
- ☐ 6
- ☐ 7

18. On average, how many minutes did you usually spend exercising at this level on one of those days?[16]

- ☐ 0
- ☐ 10
- ☐ 20
- ☐ 30
- ☐ 40
- ☐ 50
- ☐ 60

---

[13] United States, US Census Bureau. (2017). American Community Survey. Retrieved from https://www.census.gov/programs-surveys/acs/

[14] Identifying and Recommending Screening Questions for the Accountable Health Communities Model (2016, July) Technical Expert Panel discussion conducted at the U.S. Department of Health and Human Services, Centers for Medicare & Medicaid Services, Baltimore, MD.

[15] Coleman, K. J., Ngor, E., Reynolds, K., Quinn, V. P., Koebnick, C., Young, D. R., . . . Sallis, R. E. (2012). Initial Validation of an Exercise "Vital Sign" in Electronic Medical Records. Medicine and Science in Sport and Exercise, 44(11), 2071-2076. doi:10.1249/MSS.0b013e3182630ec1

[16] Ibid

- ☐ 90
- ☐ 120
- ☐ 150 or greater

Follow these 2 steps to decide if the person has a physical activity need:

1. Calculate [“number of days” selected] x [“number of minutes” selected] = [number of minutes of exercise per week]
2. Apply the right age threshold:
   - Under 6 years old: You can’t find the physical activity need for people under 6.
   - Age 6 to 17: Less than an average of 60 minutes a day shows an HRSN.
   - Age 18 or older: Less than 150 minutes a week shows an HRSN.

Substance Use

The next questions relate to your experience with alcohol, cigarettes, and other drugs. Some of the substances are prescribed by a doctor (like pain medications), but only count those if you have taken them for reasons or in doses other than prescribed. One question is about illicit or illegal drug use, but we only ask in order to identify community services that may be available to help you. [17]

19. How many times in the past 12 months have you had 5 or more drinks in a day (males) or 4 or more drinks in a day (females)? One drink is 12 ounces of beer, 5 ounces of wine, or 1.5 ounces of 80-proof spirits.

- ☐ Never
- ☐ Once or Twice
- ☐ Monthly
- ☐ Weekly
- ☐ Daily or Almost Daily

20. How many times in the past 12 months have you used tobacco products (like cigarettes, cigars, snuff, chew, electronic cigarettes)?

- ☐ Never
- ☐ Once or Twice
- ☐ Monthly
- ☐ Weekly
- ☐ Daily or Almost Daily

[17] United States, U.S. Department of Health and Human Services, National Institutes of Health. (n.d.). Helping Patients Who Drink Too Much: A Clinician's Guide (2005 ed., pp. 1-34).

21. **How many times in the past year have you used prescription drugs for non-medical reasons?**
    - ☐ Never
    - ☐ Once or Twice
    - ☐ Monthly
    - ☐ Weekly
    - ☐ Daily or Almost Daily

22. **How many times in the past year have you used illegal drugs?**
    - ☐ Never
    - ☐ Once or Twice
    - ☐ Monthly
    - ☐ Weekly
    - ☐ Daily or Almost Daily

Mental Health

23. **Over the past 2 weeks, how often have you been bothered by any of the following problems?[18]**

    a. Little interest or pleasure in doing things?
    - ☐ Not at all (0)
    - ☐ Several days (1)
    - ☐ More than half the days (2)
    - ☐ Nearly every day (3)

    b. Feeling down, depressed, or hopeless?
    - ☐ Not at all (0)
    - ☐ Several days (1)
    - ☐ More than half the days (2)
    - ☐ Nearly every day (3)

If you get 3 or more when you add the answers to questions 23a and 23b the person may have a mental health need.

---

[18] Kroenke, K., Spitzer, R. L., & Williams, J. B. (2003). The Patient Health Questionnaire-2: validity of a two-item depression screener. Medical Care, 41(11), 1284-1292.

24. Stress means a situation in which a person feels tense, restless, nervous, or anxious, or is unable to sleep at night because his or her mind is troubled all the time. Do you feel this kind of stress these days?[19]

- ☐ Not at all
- ☐ A little bit
- ☐ Somewhat
- ☐ Quite a bit
- ☐ Very much

Disabilities

25. Because of a physical, mental, or emotional condition, do you have serious difficulty concentrating, remembering, or making decisions?[20] (5 years old or older)

- ☐ Yes
- ☐ No

26. Because of a physical, mental, or emotional condition, do you have difficulty doing errands alone such as visiting a doctor's office or shopping?[21] (15 years old or older)

- ☐ Yes
- ☐ No

[19] Elo, A.L., Leppänen, A., & Jahkola, A. (2003). Validity of a Single-Item Measure of Stress Symptoms. Scandinavian Journal of Work, 29(6), 444-451.

[20] United States, U.S. Department of Health and Human Services, Office of the Assistant Secretary for Planning and Evaluation (n.d.). (2011). Implementation Guidance on Data Collection Standards for Race, Ethnicity, Sex, Primary Language, and Disability Status. Retrieved from https://aspe.hhs.gov/basic-report/hhs-implementation-guidance-data-collection-standards-race-ethnicity-sex-primary-language-and-disability-status

[21] Ibid.

Supplementary File SVI
Clues for Community Health Nursing Care Escape Room Simulation

Clue for Living Room Challenge:

Note from Mrs. Lopez:

The living room is a great place to relax and kick up my feet.
It's just getting a bit cluttered now since I'm feeling kind of beat!

This is concerning because Mr. Lopez can _ _ _ _ due to his left-sided weakness and poor vision. And if that happens to me, I'm at _ _ _ _ for fractures due to my osteoporosis.

Fill in the blanks using the words you identified above:
Count the total number of things you find in the living room that create a ________ ________.

This one-digit number goes into the lockbox first.

Clue for Kitchen Challenge:

Solve the puzzles:

1. I need to sit straight up for at least 30 minutes after I take my Fosamax (alendronate) medication or else I can get acid _ (_) _ _ _ _ as a side effect.

2. To diagnose me with pneumonia, they took an (_) - _ _ _.

3. Mr. Lopez saw an _ _ _ _ (_) _ _ _ _ _ _ _ therapist after his stroke. They helped him learn to feed and dress himself.

4. The age to be eligible for our _ _ _ (_) _ _ _ _ insurance is 65 years old.

5. Yes, we do have _ _ _ _ _ _ _ (_) _ _ _ _ _ _ to get to our appointments. But, I just wish our car didn't cost so much to maintain!

6. The _ _ (_) _ _ _ _ _ _ on the healthcare team taught us what food are best to eat for Mr. Lopez's diabetes.

7. When I prepare our food, I'm careful not to use too much (_) _ _ _ _ because Mr. Lopez is lactose intolerant.

Place the (circled) letters here in the order they appear above:
My daughters are so sweet…I sure wish they lived closer so we could go out and get something good to eat!
Count the number of _ _ _ _ _ _ _ food items the couple has due to inability to afford/access food.

This one-digit number goes into the lockbox second.

Clue for Bedroom/Bathroom Challenge:

Note from Mrs. Lopez:

Mr. Lopez takes 5 different medications every day, some twice a day.

The pharmacist is so nice and talked to me about making sure I give the correct medicines, especially after the doctor recently changed the _______ of his blood pressure medication.

But still, it's a lot. Keeping things organized is hard…there's pill bottles everywhere! You can really see how easy it can be for me to mess up his medications!

Fill in the blanks using the word you identified above:
Something's wrong here…something that can place Mr. Lopez in danger! Find the pill bottle with the incorrect ________.

This two-digit number on the pill bottle goes into the lockbox last.

Great job completing the challenges!

Here is your key to unlock the escape room!

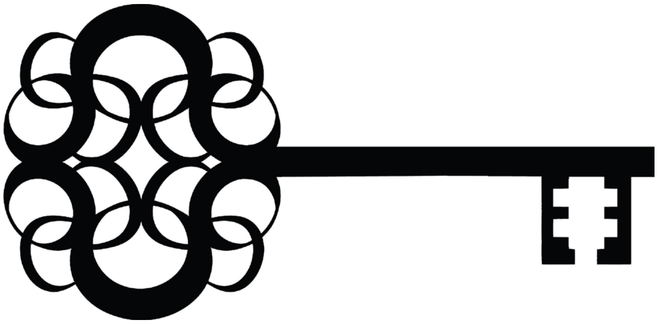

Now it is time to discuss your experience with the facilitator!

Supplementary File SVII
Escape Score Sheet for Community Health Nursing Care Escape Room Simulation

Team Name: ____________________
Date: ____________________
Faculty Observer: ____________________
Total Time to Escape: ____________________

Escape Room Completion Criteria
To successfully "escape" the room, the student team must:

- Complete the SP interview
- Complete all home/environmental assessments
- Correctly identify all required clues
- Demonstrate safe and therapeutic nursing behaviors
- Achieve minimum passing score of 80%
- Correctly enter the lockbox code sequence

Lockbox Code Score Sheet

**Challenge Area**	**Required Answer**	**Student Answer**	**Correct**
Living Room Hazard Number	_______	_______	☐
Kitchen Food Insecurity Number	_______	_______	☐
Bedroom Medication Number	_______	_______	☐

Final Lockbox Code: ___________________
☐ Successfully Escaped
☐ Did Not Escape

Section 1: Standardized Patient (SP) Interview Checklist (40 Points)

**Faculty Observation assessment Item**	**Completed**	**Points**
Introduced self and role to patient	☐	1
Explained purpose of home visit	☐	1
Demonstrated therapeutic communication	☐	2
Maintained respectful and professional behavior	☐	2
Used active listening and empathy	☐	2
Asked about current health concerns	☐	2
Asked about chronic illnesses/health conditions	☐	2
Assessed medication management	☐	2
Asked about medication adherence/problems	☐	2
Asked about caregiver responsibilities/stress	☐	2
Assessed activities of daily living (ADLs)	☐	2
Assessed mobility/fall concerns	☐	2
Asked about food access/food insecurity	☐	2
Asked about financial concerns	☐	2
Asked about transportation barriers	☐	2
Asked about social support/family support	☐	2
Assessed psychosocial/emotional stressors	☐	2
Identified safety concerns discussed by SP	☐	2

Demonstrated patient-centered communication	☐	2
Team collaborated effectively during interview	☐	2
Maintained organization and flow of interview	☐	2
Summarized findings and clarified concerns	☐	2

Section 1 Total: ______ / 40

Section 2: Home Assessment and Escape Room Tasks (48 points)

Living Room Assessment

**Assessment Item**	**Completed**	**Score**
Identified clutter/trip hazards	☐	2
Identified fall risk related to stroke/vision	☐	2
Identified osteoporosis fracture risk	☐	3
Correctly counted hazards/clue	☐	3

Subtotal: ______ / 10

Kitchen Assessment

**Assessment Item**	**Completed**	**Score**
Identified food insecurity	☐	2
Identified dietary concerns related to diabetes	☐	2
Identified transportation/access barriers	☐	2
Correctly solved medication/healthcare clues	☐	2
Correctly counted unhealthy food items	☐	2

Subtotal: ______ / 10

Bedroom/Bathroom Assessment

**Assessment Item**	**Completed**	**Score**
Identified medication safety concerns	☐	2
Identified incorrect medication dosage	☐	2
Identified medication organization issues	☐	2
Identified bathroom safety hazards	☐	2
Identified accessibility concerns	☐	2

Subtotal: ______ / 10

Comprehensive Environmental Assessment

**Assessment Item**	**Completed**	**Score**
Assessed all rooms systematically	☐	2
Identified safety hazards	☐	2
Identified chronic disease management concerns	☐	2
Prioritized patient safety appropriately	☐	2
Applied community health nursing concepts	☐	2

Subtotal: ______ / 10

Final Score Calculation

**Section**	**Points Earned**
SP Interview	______ / 40
Home Assessment	______ / 40

TOTAL SCORE: ______ / 80

Supplementary File SVIII
Debrief Guide for Community Health Nursing Care Escape Room Simulation

Introduction:
Welcome nursing students to the debriefing session after completing the Community Health Nursing Care Escape Room Simulation. This session aims to reflect on your experiences, reinforce key learnings, and address any questions or concerns you may have. Please feel free to share your thoughts openly.

I. Icebreaker (5 minutes):
Begin the debrief with an icebreaker activity to create a comfortable atmosphere for discussion. You could ask a light, non-academic question to engage everyone.

II. Reflection on Experience (20 minutes):
- Initial Reactions: Ask each student to share their initial thoughts and feelings about the escape room experience. Encourage them to be specific.
- Collaborative Efforts: Discuss the teamwork and communication dynamics within your groups. What strategies worked well, and what could be improved?
- Cultural Competency:
  - How did the escape room simulation challenge your understanding of cultural diversity and its implications in community health nursing (CHN)?
  - What cultural aspects did you encounter during the simulation that you found challenging or unfamiliar? How did you address them?
- Humility:
  - Describe a moment during the escape room simulation where you had to step back and recognize your own cultural biases or assumptions. How did this awareness impact your approach to the scenario?
  - How did you respond to situations in the escape room where your initial assumptions about a cultural practice or belief were challenged?
  - Reflect on any instances where you had to seek guidance or assistance from your peers or facilitators during the simulation. How did this demonstrate humility in practice?
- Clinical Judgment: Describe a scenario from the escape room where you had to balance clinical protocols with cultural considerations. How did you approach this situation, and what factors influenced your decision-making process?

III. Application of Community Nursing Concepts, Critical Thinking and Problem-Solving (15 minutes):
- Identifying Learning Points: Reflect on any insights or lessons learned from the escape room experience that you believe will positively impact your clinical judgment in future nursing practice, particularly in community health settings.
- How has your perspective on cultural competency in nursing changed as a result of participating in the escape room simulation?
- How did the escape room simulation enhance your ability to make culturally sensitive clinical judgments when interacting with families from diverse cultural backgrounds?
- Identify specific strategies or resources that you intend to utilize to continue developing your cultural competency, humility, and clinical judgment as a nurse.
- What steps will you take to ensure ongoing reflection and growth in these areas throughout your nursing career?
- Problem-Solving Strategies: Discuss the different problem-solving strategies employed by various groups. Were there any unique approaches that stood out?

IV. Feedback, Suggestions and Final Thoughts (5 minutes):

- Strengths and Areas for Improvement: Invite students to share their feedback on the escape room simulation. What aspects did they find particularly effective, and are there any suggestions for enhancement?
- Next Steps: Provide information on any follow-up activities, assignments, or resources related to the community nursing care module.

Supplementary File SIX
Community Health Nursing Competency Checklist

This form lists the required clinical skills for a community health nurse to provide safe, competent care at community settings. Please respond whether or not you are competent on each clinical skill by Yes or No.

**CHN competency skills**	**Y/N***
1. Demonstrate competencies of community health nursing in clinical practice at assigned setting.	
2. Assume roles and functions of community health nurse.	
3. Conduct a community assessment of a specific community.	
4. Incorporate diverse cultural beliefs and practices into assessments and interventions.	
5. Discuss community strengths and potential needs using assessment data.	
6. Determine relevant information needed	
7. Identify appropriate sources for data collection	
8. Discuss community strengths and potential needs using assessment data	
9. Incorporate data from client, family/support persons and healthcare team members	
10. Develop plan of care based on analysis of assessment data	
11. Communicate priorities and rationale for decisions to instructor	
12. Incorporate diverse cultural beliefs and practices into assessment and interventions	
13.Describe how diverse cultural, ethnic and social backgrounds function as sources of family and community values	
14. Communicate observations or concerns related to hazards to families or the community	
15. Integrate theory from nursing, natural and social sciences to enhance client care	
16. Identify social determinants of health barriers/disparities for a client of different ethnicity than you	
17. Communicate effectively related to diversity, equity, and inclusion with clients and client families	
18. Provide safe and culturally competent care in the community setting	
Total Score	

Y = Yes = 1; N = No = 0. The total score is the sum of the number of "Yes" responses.

Supplementary File SX
Community Health Nursing Confidence Scale

This form lists different activities and outcomes for this course. In the column **Confidence**, rate how confident you are that you can do them **as of now**. Rate your degree of confidence by recording a number from 0 to 100 using the scale given below:

	0	10	20	30	40	50	60	70	80	90	100
	Cannot do at all				Moderately certain can do				Highly certain can do		
1. Demonstrate competencies of community health nursing in clinical practice at assigned setting.											
2. Assume roles and functions of community health nurse.											
3. Conduct a community assessment of a specific community.											
4. Incorporate diverse cultural beliefs and practices into assessments and interventions.											
5. Discuss community strengths and potential needs using assessment data.											
6. Determine relevant information needed											
7. Identify appropriate sources for data collection											
8. Discuss community strengths and potential needs using assessment data											
9. Incorporate data from client, family/support persons and healthcare team members											
10. Develop plan of care based on analysis of assessment data											
11. Communicate priorities and rationale for decisions to instructor											
12. Incorporate diverse cultural beliefs and practices into assessment and interventions											
13.Describe how diverse cultural, ethnic and social backgrounds function as sources of family and community values											
14. Communicate observations or concerns related to hazards to families or the community											
15. Integrate theory from nursing, natural and social sciences to enhance client care											

16. Identify social determinants of health barriers/disparities for a client of different ethnicity than you	
17. Communicate effectively related to diversity, equity, and inclusion with clients and client families	
18. Provide safe and culturally competent care in the community setting	

Supplementary File SXI
The Escape Room Experience Perception Scale

Please rate your experience on the following statements using the scale provided below.

A. Strongly Disagree
B. Disagree
C. Neutral
D. Agree
E. Strongly Agree

1. My escape game experience was enjoyable.
2. The escape room enhanced my understanding of community nursing care.
3. The escape room encouraged critical thinking and problem-solving skills.
4. I felt engaged and motivated throughout the escape room activity.
5. I felt less stressed and anxious in the escape room than in the real clinic.
6. The escape room was a valuable learning experience for community health concepts.
6. The escape room was well-organized and facilitated my learning.
7. I would recommend using escape rooms for future community nursing education.
8. I was exposed to the patient cases I don't often have access to in the community clinics.
9. The escape room increased my confidence in applying community nursing principles.
10. The escape room experience helped improve my competence in for caring diverse client populations.
11. The escape room activities improve my ability to establish therapeutic relationships with diverse clients.
12. Overall, how satisfied were you with the community nursing escape room experience?

Additional Comments (Optional):
